# Effects of probiotic supplementation on major cardiovascular-related parameters in patients with type-2 diabetes mellitus: a secondary-data analysis of a randomized double-blind controlled trial

**DOI:** 10.1186/s13098-022-00822-z

**Published:** 2022-04-13

**Authors:** Fatemeh Ahmadian, Elham Razmpoosh, Hanieh-Sadat Ejtahed, Maryam Javadi, Parvin Mirmiran, Fereidoun Azizi

**Affiliations:** 1 Department of Clinical Nutrition and Dietetics, Faculty of Nutrition Sciences and Food Technology, National Nutrition and Food Technology Research Institute, Shahid Beheshti University of Medical Sciences, Tehran, Iran; 2 Nutrition and Endocrine Research Center, Research Institute for Endocrine Sciences, Shahid Beheshti University of Medical Sciences, Tehran, Iran; 3 Obesity and Eating Habits Research Center, Endocrinology and Metabolism Clinical Sciences Institute, Tehran University of Medical Sciences, Tehran, Iran; 4 Children Growth and Development Research Center, Research Institute for Prevention of Non-Communicable Diseases, Qazvin University of Medical Sciences, Qazvin, Iran; 5 Endocrine Research Center, Research Institute for Endocrine Sciences, Shahid Beheshti University of Medical Sciences, Tehran, Iran

**Keywords:** Probiotics, Diabetes mellitus, Type 2, Cardiovascular diseases, Oxidative stress, Blood pressure, Atherogenic index of plasma, Randomized controlled trial

## Abstract

**Background:**

Patients with type-2 diabetes mellitus (T2DM), have a higher risk of future cardiovascular diseases (CVD). Meanwhile, probiotics are shown to positively impact CVD-related parameters. This randomized controlled trial sought to evaluate the effects of probiotic supplementation on fundamental CVD-related parameters including atherogenic index of plasma (AIPs), blood pressure, the Framingham risk score, and antioxidant markers in patients with T2DM.

**Methods:**

Eligible participants were randomly assigned to receive 2 capsules/day of probiotics [each containing 500 mg of *L.acidophilus*(5 × 10^10^ CFU/g), *L.plantarum*(1.5 × 10^10^ CFU/g), *L.fermentum*(7 × 10^9^ CFU/g), *L.Gasseri*(2 × 10^10^ CFU/g) and 38.5 mg of fructo-oligo-saccharides], or placebo for 6 weeks. Systolic and diastolic blood pressures (SBP and DBP, respectively), mean arterial blood pressure (MAP), atherogenic indices (the ratios of TC/HDL-C, LDL-C/HDL-C, and logTG/HDL-C), the 10-year Framingham CVD risk score, as well as total anti-oxidant capacity (TAC), paraoxonase (PON) and total oxidant status (TOS) were evaluated before and after the study. Final analyses were adjusted based on baseline parameters, and potential covariates including age, sex, PUFA and sodium intakes.

**Results:**

Sixty participants completed the study. Compared with placebo, probiotic supplementation resulted in a significant decrease in SBP[-9.24 mmHg(− 14.5, − 3.9)], DBP[− 3.71 mmHg(− 6.59, − 0.83)], MAP[− 5.55 mmHg(− 8.8, − 2.31)], the Framingham risk categories [medium–low(1.5) *vs.* 2 (medium)] and logTG/HDL-C ratio [− 0.08 (− 0.14, 0)] (All *P* < 0.05) at the end of the study. No significant changes were observed in the antioxidant markers.

**Conclusion:**

Overall, probiotic supplementation for 6 weeks led to a significant improvement in major CVD-related parameters in populations with T2DM, suggesting the possible beneficial role of probiotics in lowering the risk of future CVDs associated with diabetes. Nevertheless, more studies are needed to confirm the veracity of these results.

*Trial registration*: IRCT2013100714925N1 (registered on November, 9th, 2013).

## Introduction

Type-2 diabetes mellitus (T2DM) is one of the most common metabolic diseases in humans, with a growing prevalence. The number of patients with T2DM is expected to increase to 629 million people by 2045 [1]. It is well-known that T2DM is associated with adverse effects, including cardiovascular diseases (CVD). According to a meta-analysis, there are clustering risk factors in patients with T2DM that make them have a two-to four-fold excess risk of incident coronary heart disease [2]. Evidence has demonstrated that oxidative stress plays a major role in the progression of atherosclerosis and susceptibility to CVD [3]. Plenty of studies have pointed out that T2DM is strongly associated with a decreased status of antioxidant markers, of which the major ones are paraoxonase (PON), total antioxidant capacity (TAC) and total oxidant status (TOS) parameters [4]. In fact, increased oxidative status and decreased levels of antioxidant markers in patients with T2DM are associated with insulin resistance and carotid intima-media thickness, which all concurrently contribute to the elevated CVD risk in this population [5, 6]. On the other hand, atherogenic indexes of plasma (AIPs) have been proposed to be one of the strongest predictive indicators of atherosclerosis and CVDs [7]. These new comprehensive lipid indices generally include the ratios of logarithm of triglycerides (TG)/high-density lipoprotein cholesterol (HDL-C), total cholesterol (TC)/HDL-C and low-density lipoprotein cholesterol (LDL-C)/HDL-C [8]. The prevalence of CVD in populations with T2DM has been shown to be significantly higher in patients with higher AIPs [9]. Another substantial predictor of CVDs is the Framingham score, which is the most widely used risk score that help clinicians assess the 10-year risk of developing CVDs following fundamental information of every individual [10]. According to a recent Asian population-based study, patients with T2DM have a 20-percent increased 10-year CVD risk based on the Framingham risk score, mostly due to the untreated elevated systolic blood pressure (SBP) [11].

Meanwhile, the beneficial role of probiotics in health improvement has been increasingly taken into consideration [12]. The two main strains of probiotics used for health advantages include *Lactobacillus* and *Bifidobacterium*. Studies suggest that these two strains exert positive effects on alleviating diabetes-associated complications through lowering serum cholesterol, producing short chain fatty acids, and increasing bile salt deconjugation [13, 14]. According to animal studies, *Lactobacillus* and *Bifidobacterium* strains inhibit β-cells destruction in the islets of Langerhans and result in an improvement in insulin-binding potential [15].

However, according to previous human investigations, there are inconsistent findings regarding the positive role of probiotics in the management of CVD-related markers in patients with T2DM [16–18]. A recent systematic review and meta-analysis suggested that supplementation with probiotics resulted in a significant reduction in all lipid profile parameters and SBP, but did not have a meaningful effect on diastolic blood pressure (DBP) in patients with metabolic syndrome [19]. In contrast, another meta-analysis of human studies reported that although probiotic intervention had a significant effect on both SBP and DBP measures, it did not affect the LDL-C concentration in patients with T2DM [20]. In a randomized controlled trial study, probiotic intervention was shown to have a significant effect on TAC measures in patients with T2DM [21], while other studies found no significant effects [17, 18]. Our previous findings also showed a significant improvement in serum glucose and HDL-C measures following probiotic supplementation, while no significant changes were seen in the levels of serum TC [22]. No randomized-controlled trial studies (RCTs) reported the effects of probiotics on Framingham risk score in this population, while a few human investigations evaluated the effects of probiotic supplementation on atherogenic indices, reporting both significant [23] and insignificant [24] findings. These contradictory results highlight the significance of conducting additional research on the impact of probiotics on CVD-related markers in populations with T2DM.

Hence, the present study aimed to investigate the effects of probiotic supplementation on antioxidant markers including TAC, TOS and PON, blood pressure, AIPs and the 10-year Framingham risk score, as fundamental predictors of CVDs, in patients with T2DM who have a pronounced risk of future CVD.

## Materials and methods

### Participants

This randomized controlled trial was conducted in Taleghani hospital, Tehran, Iran with the registration code IRCT2013100714925N1 (retrospectively registered on November 9th, 2013) (available at: https://en.irct.ir/trial/14380) [25]. This is a secondary data-analysis of a previous investigation [22].

Inclusion criteria were men and women aged 30–75 years who were diagnosed with T2DM according to American Diabetes Association guidelines [26]. Participants were not included if they required insulin injections, consumed probiotic supplements in the three months prior to the study initiation, or were diagnosed with kidney, liver, inflammatory, intestinal, or heart diseases, or had pulmonary and immunodeficiency disorders. In addition, individuals with pregnancy/breast-feeding, allergy diseases, and short bowel syndrome were not included.

According to a previous study [27], the sample size was determined based on glycemic profile variables. For an expected change of 15 mg/dl between the intervention and control groups and considering α = 0.05 and a power of 80%, the required sample size per group was estimated to be 30 participants. This number was increased to 34 per group to accommodate the anticipated dropout rate.

### Study design

In this double-blind RCT study, a total of 68 patients with T2DM were randomly assigned into two groups to receive either probiotic supplements (n = 34) or placebo (n = 34) for 6 weeks. Randomization was performed using a block randomization process with matched participants in each block based on their sex. Allocation concealment was performed using sealed envelopes.

Each group consumed either two placebo capsules or two probiotic capsules per day for 6 weeks. Probiotic and placebo capsules had the same appearance, and neither the patients nor the researchers were aware of the treatment in the investigation. Participants were asked not to alter their usual dietary habits and routine physical activity and to avoid consuming any other synbiotic and probiotic products or dietary supplements during the study. Compliance with the consumption was monitored by a telephone interview once a week, as well as a face-to-face interview every 2 weeks. Participants were excluded if they had taken less than 90% of the supplements or showed any gastro-intestinal adverse effects. Demographic information, food consumption and fasting blood samples were collected at the baseline and at the end of the study.

### Data collection

Macro- and micronutrient intakes were calculated using a 3 day 24-h dietary recall, with data analyzed using Nutritionist 4 software (First Databank, San Bruno, CA, USA), and household measurements used to convert the records to grams per day [28]. Physical activity level was assessed using a physical activity questionnaire (MAQ). The reliability and validity of the questionnaire were confirmed in the Iranian urban adult population [29].

The weight of the participants was measured by a weight scale (Seca, Hamburg, Germany) with 0.1-kg accuracy with minimal clothing and no shoes. Height was measured using a stadiometer (Seca) with 0.1 cm of accuracy without shoes. Body mass index (BMI) was calculated by dividing a person's weight in kilograms by their height in meters squared.

### Laboratory data

Blood samples were collected from the antecubital vein of every participant’s left arm after a 12-h overnight fast. The serum samples were separated from the whole blood by centrifugation at 3500 rpm for 10 min (Hettich D-78532; Tuttlingen, Germany). The samples were frozen immediately and stored at − 70 °C until the analysis, at the research institute for endocrine sciences, Shahid Beheshti University of Medical Sciences, Tehran, Iran.

Serum TAC, and TOS were measured by the chemical colorimetric technique using the Zell bio kit (Germany), and PON was measured by an enzymatic colorimetric method using the Zell bio kit (Germany).

The values of AIPs, including the ratios of TC/HDL-C, LDL-C/HDL-C, and log TG/HDL-C, were calculated based on previously reported data [22].

Blood pressure, including SBP and DBP, and heart rate (HR) measures were assessed using a mercury sphygmomanometer (Rossmax, Swiss, model GB-102). Mean arterial blood pressure (MAP) was calculated using a formula in which DBP was doubled and added to SBP and that composite sum was then divided by 3 [30].

The modified Framingham risk score was calculated using major coronary risk factors. The diagnosis of T2DM is a substantial risk factor for the Framingham risk score calculation. Other risk factors include sex, age, SBP, TC, HDL-C, and smoking status, as well as the treatment status of high blood pressure, and final scores were shown as percentages. Every risk factor was classified into specific categories, and each category was given a specific point [31]. The cutoffs for every risk factor were as follows: SBP: < 120, 120–129, 130–139, 140–159, and ≥ 160 mmHg; for TC < 160, 160–199, 200–239, 240–279, and  ≥ 280 mg/dL; and for HDL-C: < 40, 40–49, 50–59, and ≥ 60 mg/dL. The sum of the points was used to estimate the ten-year Framingham risk percentage, which was as follows: 1 point 6%, 2 points 8%, 3 points 10%, 4 points 12%, 5 points 16%, 6 points 20%, 7 points 25%, 10 points or more > 30%. Finally, participants were classified as having a low (10% score), moderate (10–19% score), or high (> 19% score) risk of future 10 year CVDs based on their final risk score [32].

### Ethical approval

The study was approved by the ethics committee at Shahid Beheshti University of Medical Sciences (Code: IR.SBMU.ENDOCRINE.REC.1395.250). All participants signed an informed consent form prior to the study. The trial has been registered with the Iranian registry of clinical trials, IRCT 2013100714925N1 [33].

### Intervention

Patients in the intervention group received 2 probiotic capsules per day, one after lunch and one after the evening meal, for 6 weeks. Probiotics were kindly produced by Familact Co. [34]. Participants were asked to keep the capsules in the refrigerator during the study. Each capsule contained 500 mg of probiotics which consisted of the following 7 viable and freeze-dried bacterial species: *Lactobacillus acidophilus [2 × 10*^*9*^ colony forming units (*CFU]), L_casei (7 × 10*^*9*^* CFU), L_bulgaricus (2 × 10*^*8*^* CFU), L_rhamnosus (1.5 × 10*^*9*^* CFU), Bifidobacterium breve (3 × 10*^*10*^* CFU), B. longum (7 × 10*^*9*^* CFU), Streptococcus thermophilus (1.5 × 10*^*9*^* CFU)*, and 100 mg of fructo-oligosaccharide with lactose as carrier substances*.* Patients in the placebo group received 2 placebo capsules per day, which contained the same contents with magnesium stearate and without the bacteria species.

### Statistical analysis

Statistical analyses were performed using SPSS version 21. Continuous variables were expressed as mean ± standard deviation (SDs) and descriptive statistics were presented as frequencies. Chi-square tests and independent sample t-tests were used to compare the qualitative and quantitative variables between the two groups at the beginning of the study. For between-group comparisons of non-normal quantitative variables, Mann–Whitney U tests were used.

The normality of the distribution of variables was checked by the Kolmogorov–Smirnov test. The log-transformation was used for the variables that were non-normally distributed.

The paired sample t-tests and Wilcoxon Signed Ranks tests were applied to compare the mean values of variables before and after the intervention in each group. Finally, differences between the two groups after the intervention were specified by the analysis of covariance (ANCOVA), adjusting for baseline measurements, age, sex, polyunsaturated fatty acids (PUFA), and sodium intakes as the confounding factors. The Chi-square test and Friedman test were used for between-group and within-group differences in the categorical variables at the end of the study.

## Results

**Fig. 1:**
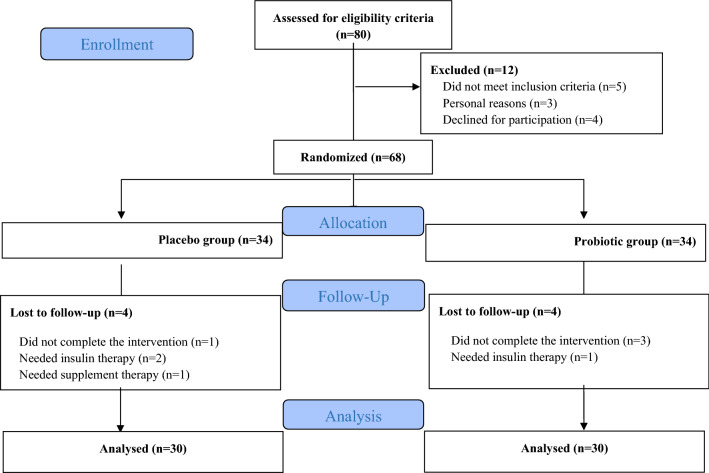
Flow of study

The initial participants of the study included a total of 68 patients with T2DM. Eight participants were excluded from the statistical analysis (4 individuals in each of the probiotic and placebo groups) since they needed to have either insulin (n = 3) or supplement therapy (n = 1) or they did not complete their interventions at the expected time (n = 4). Finally, data was analyzed for 60 participants (30 individuals in each group). Figure 1 shows the complete flow of the study based on the Consolidated Standards of Reporting Trials (CONSORT) flow diagram.

Patients did not report any adverse effects related to the probiotic consumption during the study. Table 1 shows the characteristics of the participants in each group at baseline.

**Table 1:** Baseline characteristics of participants

	Probiotic group (n = 30)	Placebo group (n = 30)	*P*-value*
Age (y)	58.5 (52–64)	61 (57–65)	**0.018**
Sex (male, %)	17 (56.7%)	16 (53.3%)	0.36
Weight (kg)	75.24 ± 16	74.1 ± 9	0.79
BMI^a^ (kg/m^2^)	27.4 ± 4	27.16 ± 4	0.63
Physical activity			
Low	11 (36.7%)	13 (44.8%)	0.52
Moderate	19 (63.3%)	16 (55.2%)	
Drug consumption			
Metformin	14 (46.7%)	11 (36.7%)	0.9
Others	16 (53.3%)	19 (63.3%)	
Duration of diabetes (y)	6.16 ± 3	5.89 ± 3	0.72

Bold indicates the significant measures

Age is presented as median and interquartile range

Data are presented as mean ± SD for continuous and percent for categorical variables

^*^P value obtained from either one sample t-test or chi-square test

^a^BMI, body mass index

After the final analyses, a statistically significant difference was observed between the two groups with respect to the mean age of patients (*P* = 0.018), while other baseline characteristics of the patients did not differ between the two groups (*P* > 0.05). The analysis of dietary intakes is shown in Table 2. At the beginning of the study, no significant differences were found between the two groups regarding energy, carbohydrate, protein, saturated fatty acid, monounsaturated fatty acid, cholesterol, dietary fiber and vitamin C intakes (*P* > 0.05). However, there were significant differences between the two groups with respect to the intake of PUFA, and sodium intakes at the baseline of the study (*P* < 0.05). In addition, considerable differences in dietary intakes of energy, saturated fatty acids, monounsaturated fatty acids, and dietary fiber were found between the two groups, at the end of the study (*P* < 0.05). At the baseline, no significant differences were found between the two groups in terms of blood pressure, AIPs, the Framingham risk score and the antioxidant biomarkers including TAC, TOS and PON (*P* > 0.05).

**Table 2:** Dietary intakes of participants throughout the study

Variables	Probiotic group (n = 30)	Placebo group (n = 30)	*P*-value
Energy (Kcal)			
Baseline	1966 ± 454	2009 ± 415	0.7
After intervention	1851 ± 309	2044 ± 378	**0.03**^**‡**^
* P*^†^	0.12	0.54	
Carbohydrate (g)			
Baseline	242 ± 64.4	242 ± 65.2	0.61
After intervention	228 ± 46	256 ± 73	0.09
* P*^†^	0.14	0.18	
Protein (g)			
Baseline	80 ± 37	73 ± 25	0.4
After intervention	74 ± 26	83 ± 36	0.2
* P*^†^	0.47	0.19	
Total fat (g)			
Baseline	74.4 ± 35	85.3 ± 25	0.17
After intervention	72.1 ± 24	81.1 ± 22	0.16
* P*^†^	0.68	0.44	
Saturated fat (g)			
Baseline	20 ± 8	21 ± 7	0.9
After intervention	18 ± 5	22.5 ± 16	**0.04**^**‡**^
* P*^†^	0.13	0.18	
Monounsaturated fat (g)			
Baseline	18.22 ± 8.95	20 ± 8	0.36
After intervention	15.86 ± 6.35	20 ± 7	**0.02**^**‡**^
* P*^†^	0.74	0.89	
Polyunsaturated fat(g)			
Baseline	24 ± 24	38.9 ± 23	0.011^*^
After intervention	30 ± 20	33 ± 14	0.5
* P*^†^	0.28	0.11	
Cholesterol			
Baseline	208 ± 145	195 ± 108	0.7
After intervention	209 ± 141	208 ± 139	0.9
* P*^†^	0.95	0.72	
Dietary fiber (g)			
Baseline	9.6 ± 5.4	10.6 ± 5	0.49
After intervention	7.5 ± 4.9	10.6 ± 4	**0.008**^**‡**^
* P*^†^	0.003	0.59	
Vitamin E			
Baseline	15 ± 12	16.8 ± 13	0.29
After intervention	17.9 ± 15	17 ± 8	0.79
* P*^†^	0.1	0.48	
Vitamin C			
Baseline	59 ± 50	56 ± 50	0.8
After intervention	82 ± 97	83 ± 93	0.9
* P*^†^	0.18	0.12	
Calcium (mg)			
Baseline	774.9 ± 246.4	740.1 ± 313.8	0.64
After intervention	729.8 ± 267.9	875.6 ± 342.5	0.07
* P*^†^	0.42	0.013	
Magnesium (mg)			
Baseline	248.2 ± 62.7	240.7 ± 66.3	0.66
After intervention	250.1 ± 58	251.6 ± 61.1	0.92
* P*^†^	0.9	0.42	
Sodium (mg)			
Baseline	2407.3 ± 998.7	1834.2 ± 868.9	**0.02**
After intervention	2049.7 ± 782.2	2314 ± 794.7	0.19
* P*^†^	0.036	0.001	

Data are presented as mean ± SD

^*^P value obtained from independent samples t-test

^†^*P* value obtained from paired sample t-test

^‡^Analysis of covariance, adjusted for age, sex, difference intake of polyunsaturated fatty acid and sodium, and baseline values

Results from the final analyses indicated that there were significant differences between the two groups in the levels of SBP, DBP and MAP as well as the measures of the Framingham risk categories and the ratio of logTG/HDL-C at the end of the study in the intervention group after adjusting for baseline measures and covariates, compared to the placebo group (*P* < 0.05). There were no considerable differences in the levels of other parameters between the two groups at the end of the study. Within-group analyses indicated that there was a significant decrease in the measures of SBP, DBP, MAP, logTG/HDL-C and the Framingham risk score in the probiotic group. The measures of DBP and MAP were also decreased significantly in the control group, though with a smaller median difference (Table 3).

**Table 3:** Changes in cardiovascular-related parameters at the baseline and after the 6-week probiotic intervention

Variable	Period	Probiotic group (n = 30)	Placebo group (n = 29)	MD (95% CI)† between groups	*P* value
TAC (mmol/L)	Initial	0.28 (0.06)	0.28 (0.05)	0 (−0.02, 0.03)	0.8
	End	0.3 (0.08)	0.28 (0.06)	0.02 (−0.02, 0.04)	0.42
	MD (95% CI) within groups*	0.02 (0, 0.03)	0 (−0.01, 0.01)		
	*P* value	0.1	0.94		
TOS (µmol/L)	Initial	2.45 (2.13, 3.75)	2.47 (2, 3.26)	−0.02	0.85
	End	2.23 (2.01, 2.46)	2.26 (2.26, 3.52)	−0.03	0.53
	Median difference	−0.22	−0.21		
	*P* value	0.16	0.68		
PON (mmol/L)	Initial	93.65 (65.87, 189.87)	83.35 (64, 148)	10.3	0.64
	End	83.75 (66.47, 210.17)	86.55 (59.57, 153.32)	−2.8	0.09
	Median difference	−9.9	3.2		
	*P* value	0.46	0.31		
SBP (mmHg)	Initial	120.63 (11.56)	117.01 (12.25)	3.63 (−2.5, 9.8)	0.25
	End	109.2 (8.66)	118.45 (11.46)	−9.24 (−14.5, −3.9)	** < 0.001**
	MD (95% CI) within groups*	−11.43 (−13.8, −9)	1.44 (−0.87, 3.7)		
	*P* value	** < 0.001**	0.21		
DBP (mmHg)	Initial	80.63 (3.41)	79.38 (2.54)	1.25 (−0.32, 2.8)	0.12
	End	70.7 (4.8)	74.41 (6.17)	−3.71 (−6.59, −0.83)	**0.007**
	MD (95% CI) within groups*	−9.9 (−12.09, −7.75)	−4.98 6 (−2.8, 4.24)		
	*P* value	** < 0.001**	**0.01**		
HR (n/min)	Initial	75.4 (11.8)	80.48 (9.17)	−5.08 (−11.35, 1.19)	0.11
	End	76.9 (11.38)	81.2 (8.9)	−4.3 (−10.38, 1.77)	0.38
	MD (95% CI) within groups*	1.5 (0, 3.9)	0.72 (−2.8, 4.2)		
	*P* value	0.22	0.67		
MAP (mmHg)	Initial	93.96 (4.78)	91.11 (4.85)	2.04 (−0.52, 4.61)	0.12
	End	83.53 (5.31)	87.29 (6.69)	−5.55 (−8.8, −2.31)	** < 0.001**
	MD (95% CI) within groups*	−10.43 (−12.04, −8.7)	−3.8 (−5.85, −1.7)		
	*P* value	** < 0.001**	**0.01**		
Framingham Score categories (n, %)ǂ	Initial			−1.3	0.77
	Low	7 (23.3)	4 (13.8)		
	Medium	15 (50)	16 (55.2)		
	High	8 (26.7)	9 (31)		
	End			–	**0.032**
	Low	18 (60)	10 (34.5)		
	Medium	10 (33.3)	13 (44.8)		
	High	2 (6.7)	6 (20.7)		
	Median difference	–	–	–	
	*P* value	< **0.001**	0.052		
TC/HDL-C	Initial	3.32 (2.13, 3.75)	3.23 (2.79, 3.58)	0.09	0.69
	End	2.23 (2.01, 2.46)	2.97 (2.81, 4)	−0.74	0.55
	Median difference	−1.09	−0.35		
	*P* value	0.16	0.96		
LDL-C/HDL-C	Initial	1.93 (1.17)	1.66 (0.75)	0.04 (−0.47, 0.57)	0.85
	End	1.78 (0.89)	1.67 (0.79)	−0.11 (−0.57, 0.36)	0.53
	MD(95% CI) within groups*	−0.15 (−0.36, 0.05)	0.02 (-0.22, 0.21)		
	*P* value	0.15	0.87		
logTG/HDL-C	Initial	0.49 (0.27)	0.47 (0.17)	0.02 (−0.1, 0.13)	0.78
	End	0.4 (0.14)	0.48 (0.11)	−0.08 (−0.14, 0)	**0.023**
	MD(95% CI) within groups*	−0.09 (−0.15, 0)	0.01 (−0.05, 0.07)		
	*P* value	**0.04**	0.72		

Data are shown as mean (SD); data for Framingham score, TOS, PON and TC/HDL-C are presented as median (25, 75 percentiles)

*MD* mean differences, *TAC* total anti-oxidant capacity, *TOS* total oxidant status, *PON* paraoxonases, *SBP* systolic blood pressure, *DBP* diastolic blood pressure, *HR* heart rate, *MAP* mean arterial pressure, *TC* total cholesterol, *HDL-C* high density of lipoprotein cholesterol, *LDL-C* low-density of lipoprotein cholesterol, *TG* triglycerides

^*^Paired student t-tests were used for within-group comparisons, except for Framingham score, TOS, PON and TC/HDL-C measures which was estimated by non-parametric Wilcoxon signed ranks tests

^**†**^Independent student t-tests were used at the beginning of the study for between-group comparison, except Framingham score, TOS, PON and TC/HDL-C measures which was estimated by Mann–Whitney U-tests. At the end of the study, differences between groups were assessed using analysis of covariance (ANCOVA) adjusted for baseline values, age, BMI and energy intake

ǂChi-square test and Friedman test were used for between-group and within-group differences

## Discussion

The present investigation aimed to assess the effect of probiotics on major predictors of CVDs, including antioxidant parameters, AIPs, blood pressure, and the 10-year Framingham risk score in patients with T2DM. The key findings indicated that patients who consumed probiotics for 6 weeks had lower levels of SBP, DBP, MAP, logTG-HDL-C, and Framingham risk categories compared to the placebo group, after adjusting for baseline parameters and potential confounders.

In line with our results, a systematic review and meta-analysis of 14 studies showed that probiotics and synbiotics supplementation had a significant reducing effect on both SBP and DBP levels in populations with T2DM [16]. In fact, it has been hypothesized that the angiotensin-converting enzyme inhibitors can be converted into active form through the fermentation process by probiotic bacteria, which positively affects SBP, DBP and MAP measures [35]. However, this effect has been found to be much higher when probiotics are taken through fermentable foods [36]. Similarly, evidence has proven that the antioxidant-increasing effects of probiotics were only seen when they were consumed in combination with fermentable foods, including dairy products, which might be mostly due to the overgrowth and elevated activities of probiotic strains [37]. That would be the main reason for not achieving the significant changes in antioxidant parameters including TAC, TOS and PON in the present study. The short-duration of the intervention and differences in ethnicity might be another reason for the present insignificant findings.

On the other hand, a few investigations have evaluated the effects of either probiotics or synbiotics on AIPs among patients with T2DM [21, 23, 24]. Sabico et al. assessed the pure intervention of probiotics for 12 weeks in participants with T2DM and, consistent with our findings, they reported no significant changes in TC/HDL-C ratio [24]. However, Ejtahed et al. evaluated the effect of yogurt enriched with probiotics on AIPs in individuals with T2DM, and similar to our results, they showed a significant decrease in logTG/HDL-C ratio [21]. It should be noted that although Ejtahed et al. reported positive effects of probiotics on AIP, they used yogurt as the probiotic carrier instead of probiotic supplements alone [21]. As mentioned above, dairy products enriched with probiotics have not only positive effects on the growth of probiotics strains, but exert promising effects on cardiovascular-related parameters because of the presence of other ingredients like calcium, sphingolipid, and protein [38]. This indicates that the efficacy of dairy products enriched with probiotics on AIPs may not be considerable enough to introduce probiotics as CVD-preventive agents, and hence, our finding regarding the sole positive effect of probiotics supplements on AIPs is noteworthy.

The present study also showed that the Framingham risk categories were reported to be significantly decreased in the intervention group compared to the placebo, following the probiotic supplementation. Although all participants with T2DM have one major component of the Framingham risk score (which is the diagnosis of T2DM), this score is still useful for future CVD risk assessment in this population [11]. To our knowledge, none of the previous human studies assessed the effect of probiotics on Framingham risk scores in patients with T2DM, and our findings regarding the possible role of probiotic supplementation in reducing the 10-year CVD risk in this population would be promising. The present beneficial findings may be predominantly due to the significant improvements in SBP measures. Besides, positive changes in HDL-C levels following the probiotic supplementation [22], would be another important cause of the improved Framingham risk scores.

The most important mechanisms behind these beneficial effects of probiotics on CVD-related parameters are that probiotics seem to be able to alleviate the inflammatory status in patients with T2DM through the production of short-chain fatty acids and by down-regulating inflammatory mediators such as NFκB [39]. Besides, the positive correlation between probiotics and serum HDL-C levels would be another important related mechanism [40]. In fact, HDL-C transports cholesterol in the form of cholesteryl esters to the liver for further hydrolysis [41], and that is why probiotics may have a hypocholesterolemic effect via altering the pathways of cholesteryl esters and lipoprotein transporters [42]. A meta-analysis also highlighted that an increase in HDL-C levels is strongly associated with a reduced risk of CVD compared with the changes in serum TG levels [43]. That would be the major reason for the present significant improvement in logTG/HDL-C as the amin atherogenic index.

The present study has many advantages. We evaluated the effects of probiotics on AIPs, blood pressure, the 10 year Framingham risk score, and the antioxidant markers, which are considered the most potential predictors of CVD risk in patients with T2DM, who have a pronounced risk of future CVD due to their disease status. We also used a high dose of probiotic-containing capsules, without any food carriers, to evaluate the exact effects of probiotics on T2DM. Moreover, we did not apply any dietary changes and we excluded participants that started insulin therapy or taking other types of supplements, in order to assess the exact effect of probiotic intervention.

However, limitations of the present study include the lower sample size and shorter duration of the intervention. Alterations in some dietary intakes of participants were also observed at the end of the study. Besides, as many complications of T2DM usually develop over a longer period of time, our 6 week clinical trial would not be enough to improve all the parameters and conditions in this population. Stool samples were not also evaluated to assess the microbial composition of the gut and feces. Furthermore, due to budget limitations, we were not able to assess other biochemical parameters related to future CVD risks in patients with T2DM.

## Conclusions

This RCT study showed that patients with T2DM who consumed probiotic capsules for 6 weeks significantly lowered SBP, DBP and MAP compared to the placebo group. Moreover, a significant decrease in logTG/HDL-C, as the atherogenic index, and a meaningful reduction in the Framingham risk categories were observed in the intervention group compared with the control, all of which may suggest a CVD-preventive effect of probiotics among patients with T2DM who are prone to higher risk of future CVDs. However, no significant changes were seen in any of the antioxidant parameters. Future studies are suggested to collect stool samples and perform correlation in modulation of microbial diversity with improved CVD-related parameters. Studies with longer duration and various supplement dosages among different ethnic groups are also needed to confirm the veracity of the CVD-preventive role of probiotics in this population.

## Data Availability

Not applicable.

## Abbreviations

T2DM: Type-2 diabetes mellitus
CVD: Cardiovascular disease
AIP: Atherogenic index of plasma
SBP: Systolic blood pressure
DBP: Diastolic blood pressures
MAP: Mean arterial blood pressure
TC: Total cholesterol
HDL-C: High density of lipoprotein cholesterol
LDL-C: Low density of lipoprotein cholesterol
TG: Triglycerides
TAC: Total anti-oxidant capacity
PON: Paraoxonase
TOS: Total oxidant status
PUFA: Poly-unsaturated fatty acids
RCT: Randomized-controlled trial
MAQ: Physical activity questionnaire
CFU: Colony forming unit
ANCOVA: Analysis of covariance
NFκB: Nuclear factor kappa-light-chain-enhancer of activated B cells

## References

[1] International Diabetes Federation. IDF Diabetes Atlas Brussels, Belgium2021 [updated 2021. 10:[Available from: http://www.diabetesatlas.org.

[2] Sarwar N, Gao P, Seshasai SR, Gobin R, Kaptoge S, Di Angelantonio E, Ingelsson E, Lawlor DA, Selvin E, Stampfer M (2010). Diabetes mellitus, fasting blood glucose concentration, and risk of vascular disease: a collaborative meta-analysis of 102 prospective studies. Lancet.

[3] Asmat U, Abad K, Ismail K (2016). Diabetes mellitus and oxidative stress-a concise review. Saudi Pharm J.

[4] Bigagli E, Lodovici M (2019). Circulating oxidative stress biomarkers in clinical studies on Type 2 diabetes and its complications. Oxid Med Cell Longev.

[5] Maritim A, Sanders A, Watkins Iii J (2003). Diabetes, oxidative stress, and antioxidants: a review. J Biochem Mol Toxicol.

[6] Gómez-Marcos MA, Recio-Rodríguez JI, Rodríguez-Sánchez E, Patino-Alonso MC, Magallón-Botaya R, Martínez-Vizcaino V, Gómez Sánchez L, García-Ortiz L (2011). Carotid intima-media thickness in diabetics and hypertensive patients. Rev Esp Cardiol.

[7] Shen S, Lu Y, Qi H, Li F, Shen Z, Wu L, Yang C, Wang L, Shui K, Wang Y (2016). Association between ideal cardiovascular health and the atherogenic index of plasma. Medicine.

[8] Li Y-W, Kao T-W, Chang P-K, Chen W-L, Wu L-W (2021). Atherogenic index of plasma as predictors for metabolic syndrome, hypertension and diabetes mellitus in Taiwan citizens: a 9-year longitudinal study. Sci Rep.

[9] Li Z, Huang Q, Sun L, Bao T, Dai Z (2018). Atherogenic index in Type 2 diabetes and its relationship with chronic microvascular complications. Int J Endocrinol.

[10] The Framingham Heart Study: Cardiovascular Disease (10-year risk). https://framinghamheartstudy.org/fhs-risk-functions/cardiovascular-disease-10-year-risk/.

[11] Mondal R, Ritu RB, Banik PC (2021). Cardiovascular risk assessment among type-2 diabetic subjects in selected areas of Bangladesh: concordance among without cholesterol-based WHO/ISH, Globorisk, and Framingham risk prediction tools. Heliyon.

[12] Aggarwal J, Swami G, Kumar M (2013). Probiotics and their effects on metabolic diseases: an update. J Clin Diagn Res.

[13] Kobyliak N, Virchenko O, Falalyeyeva T (2016). Pathophysiological role of host microbiota in the development of obesity. Nutr J.

[14] Samuel BS, Shaito A, Motoike T, Rey FE, Backhed F, Manchester JK, Hammer RE, Williams SC, Crowley J, Yanagisawa M, Gordon JI (2008). Effects of the gut microbiota on host adiposity are modulated by the short-chain fatty-acid binding G protein-coupled receptor, Gpr41. Proc Natl Acad Sci USA.

[15] Matsuzaki T, Nagata Y, Kado S, Uchida K, Kato I, Hashimoto S, Yokokura T (1997). Prevention of onset in an insulin-dependent diabetes mellitus model, NOD mice, by oral feeding of *Lactobacillus**casei*. APMIS.

[16] Kocsis T, Molnár B, Németh D, Hegyi P, Szakács Z, Bálint A, Garami A, Soós A, Márta K, Solymár M (2020). Probiotics have beneficial metabolic effects in patients with type 2 diabetes mellitus: a meta-analysis of randomized clinical trials. Sci Rep.

[17] Ghafouri A, Zarrati M, Shidfar F, Heydari I, Shokouhi Shoormasti R, Eslami O (2019). Effect of synbiotic bread containing lactic acid on glycemic indicators, biomarkers of antioxidant status and inflammation in patients with type 2 diabetes: a randomized controlled trial. Diabetol Metab Syndr.

[18] Tonucci LB, dos Olbrich Santos KM, de Licursi Oliveira L, Rocha Ribeiro SM, Duarte Martino HS (2017). Clinical application of probiotics in type 2 diabetes mellitus: a randomized, double-blind, placebo-controlled study. Clin Nutr.

[19] Arabi SM, Bahrami LS, Rahnama I, Sahebkar A (2022). Impact of synbiotic supplementation on cardiometabolic and anthropometric indices in patients with metabolic syndrome: a systematic review and meta-analysis of randomized controlled trials. Pharmacol Res.

[20] Ardeshirlarijani E, Tabatabaei-Malazy O, Mohseni S, Qorbani M, Larijani B, Baradar Jalili R (2019). Effect of probiotics supplementation on glucose and oxidative stress in type 2 diabetes mellitus: a meta-analysis of randomized trials. Daru.

[21] Ejtahed HS, Mohtadi-Nia J, Homayouni-Rad A, Niafar M, Asghari-Jafarabadi M, Mofid V, Akbarian-Moghari A (2011). Effect of probiotic yogurt containing *Lactobacillus**acidophilus* and *Bifidobacterium**lactis* on lipid profile in individuals with type 2 diabetes mellitus. J Dairy Sci.

[22] Razmpoosh E, Javadi A, Ejtahed HS, Mirmiran P, Javadi M, Yousefinejad A (2019). The effect of probiotic supplementation on glycemic control and lipid profile in patients with type 2 diabetes: a randomized placebo controlled trial. Diabetes Metab Syndr.

[23] Mazruei Arani N, Emam-Djomeh Z, Tavakolipour H, Sharafati-Chaleshtori R, Soleimani A, Asemi Z (2019). The effects of probiotic honey consumption on metabolic status in patients with diabetic nephropathy: a randomized, double-blind controlled trial. Probiot Antimicrob Proteins.

[24] Sabico S, Al-Mashharawi A, Al-Daghri NM, Yakout S, Alnaami AM, Alokail MS, McTernan PG (2017). Effects of a multi-strain probiotic supplement for 12 weeks in circulating endotoxin levels and cardiometabolic profiles of medication naïve T2DM patients: a randomized clinical trial. J Transl Med.

[25] Iranian Randomized Clinical Trial (IRCT). The effect of probiotic supplementation on glycemic control and lipid profile in patients with type 2 diabetes: a randomized placebo controlled trial. https://pubmed.ncbi.nlm.nih.gov/30641692/. 10.1016/j.dsx.2018.08.008 30641692

[26] American Diabetes A (2010). Diagnosis and classification of diabetes mellitus. Diabetes Care.

[27] Mohamadshahi M, Veissi M, Haidari F, Shahbazian H, Kaydani GA, Mohammadi F (2014). Effects of probiotic yogurt consumption on inflammatory biomarkers in patients with type 2 diabetes. Bioimpacts.

[28] Gibson AA, Hsu MS, Rangan AM, Seimon RV, Lee CM, Das A, Finch CH, Sainsbury A (2016). Accuracy of hands v. household measures as portion size estimation aids. J Nutr Sci.

[29] Momenan AA, Delshad M, Sarbazi N, Rezaei Ghaleh N, Ghanbarian A, Azizi F (2012). Reliability and validity of the Modifiable Activity Questionnaire (MAQ) in an Iranian urban adult population. Arch Iran Med.

[30] DeMers D, Wachs D. Physiology, Mean Arterial Pressure. In: *StatPearls.* Treasure Island (FL): StatPearls Publishing Copyright^©^. 2022. San Francisco: StatPearls Publishing LLC.; 2022.

[31] Framingham Risk study: Hard Coronary Heart Disease (10-year risk) 2001 [Based on the Adult Treatment Panel III, JAMA]. Available from: https://framinghamheartstudy.org/fhs-risk-functions/hard-coronary-heart-disease-10-year-risk/.

[32] Anderson TJ, Grégoire J, Pearson GJ, Barry AR, Couture P, Dawes M, Francis GA, Genest J, Grover S, Gupta M (2016). 2016 Canadian cardiovascular society guidelines for the management of dyslipidemia for the prevention of cardiovascular disease in the adult. Can J Cardiol.

[33] The effect of Familact probiotic supplementation on lipid profile, glycemic control and insulin levels in patients with type 2 diabetes. IRCT registration number: IRCT2013100714925N1. https://en.irct.ir/trial/14380.

[34] Zist Takhmir; Familact. http://zisttakhmir.com/.

[35] Seppo L, Jauhiainen T, Poussa T, Korpela R (2003). A fermented milk high in bioactive peptides has a blood pressure-lowering effect in hypertensive subjects. Am J Clin Nutr.

[36] Jauhiainen T, Rönnback M, Vapaatalo H, Wuolle K, Kautiainen H, Groop PH, Korpela R (2010). Long-term intervention with *Lactobacillus**helveticus* fermented milk reduces augmentation index in hypertensive subjects. Eur J Clin Nutr.

[37] Melini F, Melini V, Luziatelli F, Ficca AG, Ruzzi M (2019). Health-promoting components in fermented foods: an up-to-date systematic review. Nutrients.

[38] Smedman AEM, Gustafsson I-B, Berglund LGT, Vessby BOH (1999). Pentadecanoic acid in serum as a marker for intake of milk fat: relations between intake of milk fat and metabolic risk factors. Am J Clin Nutr.

[39] Diao Y, Xin Y, Zhou Y, Li N, Pan X, Qi S, Qi Z, Xu Y, Luo L, Wan H (2014). Extracellular polysaccharide from *Bacillus* sp. strain LBP32 prevents LPS-induced inflammation in RAW 264.7 macrophages by inhibiting NF-κB and MAPKs activation and ROS production. Int Immunopharmacol.

[40] Cho YA, Kim J (2015). Effect of probiotics on blood lipid concentrations: a meta-analysis of randomized controlled trials. Medicine.

[41] Kosmas CE, Martinez I, Sourlas A, Bouza KV, Campos FN, Torres V, Montan PD, Guzman E (2018). High-density lipoprotein (HDL) functionality and its relevance to atherosclerotic cardiovascular disease. Drugs Context.

[42] Pereira DI, Gibson GR (2002). Effects of consumption of probiotics and prebiotics on serum lipid levels in humans. Crit Rev Biochem Mol Biol.

[43] Berger S, Raman G, Vishwanathan R, Jacques PF, Johnson EJ (2015). Dietary cholesterol and cardiovascular disease: a systematic review and meta-analysis. Am J Clin Nutr.

